# The Application of Lexical Retrieval Training in Tablet-Based Speech-Language Intervention

**DOI:** 10.3389/fneur.2020.583246

**Published:** 2020-11-16

**Authors:** Jeanne Gallée, Rachel Pittmann, Suzanne Pennington, Sofia Vallila-Rohter

**Affiliations:** ^1^Division of Medical Sciences, Harvard University, Cambridge, MA, United States; ^2^Program in Speech and Hearing Bioscience and Technology, Harvard Medical School, Boston, MA, United States; ^3^Department of Communication Sciences and Disorders, MGH-Institute of Health Professions, Boston, MA, United States

**Keywords:** telepractice, aphasia, lexical retrieval training, effortful learning, technology

## Abstract

In the setting of shortened hospitalization periods, periods of confinement and social isolation, limited resources, and accessibility, technology can be leveraged to enhance opportunities for rehabilitative care (1). In the current manuscript, we focus on the use of tablet-based rehabilitation for individuals with aphasia, a language disorder that frequently arises post-stroke. Aphasia treatment that targets naming through effortful and errorful instances of lexical retrieval, where corrective feedback is generated on every trial, may enhance retention and generalizability of gains (2, 3). This pilot evaluation explored how six individuals with aphasia interacted with a tablet-based therapy application that targeted lexical retrieval. Participants with aphasia either (1) autonomously engaged with the therapy tasks or (2) received systematic encouragement to effortfully retrieve words. Behaviors of response latency and cue use were examined to gain insights into the behavioral patterns of both groups, as well as analyses of task accuracy and outcomes on standardized cognitive–linguistic assessments. Despite some variability, initial observations suggest that participants who received systematic training refrained from using cues to complete tasks and spent longer on each trial, which ultimately co-occurred with increased independent engagement with therapy and improved standardized outcomes. Preliminary results present an alternative means of leveraging technology to implement best-practice recommendations in the context of aphasia telerehabilitation.

## Introduction

Technology-based teletherapies for aphasia are increasingly utilized in outpatient treatment as treatment of this type bypasses barriers imposed by financial, geographical, and temporal access (4, 5). Beyond circumventing these constraints, such therapies can be leveraged to increase the intensity of treatment as they are accessible from any setting at any time (1, 6–12). Furthermore, even when therapy is administered remotely, clinicians are able to monitor progress and tailor therapy to a client's unique needs (4, 5, 8, 13–15). Recent work by Kiran et al. (16) demonstrated that utilizing tablet-based language rehabilitation can simultaneously increase the intensity of practice while also tailoring treatment to the individual participant's needs. Furthermore, work by Godlove et al. (14) suggests that the environment in which tablet-based therapy is administered (home vs. clinical context) does not impact the extent to which naming gains are incurred.

However, gaps in the literature about the behaviors that people with aphasia (PWA) develop when engaging with tablet-based therapies remain (17–20). The effectiveness of tablet-based therapy is rooted in a person's ability to integrate and learn from the individual treatment tasks; however, little is known about how individuals with aphasia go about navigating and learning from technologically based interventions independently.

Studies of lexical retrieval are increasingly evaluating cue use, success, and effort, the results of which suggest that effortful and successful lexical retrieval promote the greater gains in naming (2, 3, 21, 22). Effortful treatment conditions for aphasia provide therapy participants the opportunity to make errors and receive feedback on performance accuracy (3, 23), such as through the presentation of visuals, sounds, verbal cues, or other clinician or tablet-based actions.

Such effortful conditions are often described as errorful therapy, as individuals are made aware of their errors, and have been found to be particularly beneficial in naming rehabilitation as they call the individual to draw information from long-term memory (3, 21, 22). Furthermore, there is evidence that the practice of autonomously retrieving a stimulus name, even when incorrect, improves treatment outcomes (2, 22–25). Moreover, greater long-term retention is observed particularly in conditions of effort, consistent with the principle that repeated retrieval practice improves access to stored information (2, 3, 22). Feedback on trial accuracy is often provided in the context of errorful tablet-based therapies, but what remains to be established is how patients engage with, learn from, and manage the feedback provided by the applications.

An essential aspect of effortful, errorful, and repeated lexical treatment practice is that it calls upon the individual to self-monitor and self-correct his or her choices (26), heightening the engagement of the individual client and enhancing long-term gains. A study by Pyc and Rawson (27) found a relationship between long-term retention and increased target retrieval time, where increased response times were interpreted as a reflection of increased effort. This is consistent with the findings by Schwartz et al. (26): the more a patient self-monitors and exerts effort to retrieve a response, the more time he or she will take to give a response.

However, past literature suggests that certain PWA do not develop strategies to effectively manage feedback-based instruction (28). Cognitive deficits are increasingly being identified in aphasia (29–36) spanning the domains of attention, memory, executive function, language, and visuospatial skills (29, 33, 36–45). Impairments in these domains might influence the way that PWA independently approach treatment that taps into effortful lexical retrieval practice. For example, Villard and Kiran (35) suggest that attention, or the lack thereof, can greatly influence not only the naming performance but also the language treatment outcomes. Furthermore, the importance of the quality, in addition to the quantity of practice, cannot be overlooked in evaluating treatment (46, 47). While a frequent and well-spaced dosage of treatment is necessary for improvement (3, 12), the quality of treatment, as shaped by speech therapist input and client output (24), must be considered. As rehabilitation demands requirement for tablet-based applications to become increasingly utilized within and *as* therapy, it is essential to explore (1) the ways in which PWA independently engage with technologically based telepractice for naming rehabilitation and (2) the ways in which behavioral training can serve to improve the quality of independent practice.

Apart from being corrective, the feedback provided in tablet-based naming therapy can include the possibility of pre-response cues: visual and/or auditory features that provide further information about a particular stimulus before a response is selected. As such, users can self-administer cues to either find out or verify an answer. Des Roches et al. (19) found that there are distinct profiles of cue use in people who participate in tablet-based language treatment and that the self-administration of cues may relate to aphasia severity: more severe patients tended to use more cues. The authors' findings highlighted two dichotomized profiles of cue use, (1) participants who had higher performance accuracy with increased cue use and (2) participants who had lower performance accuracy with increased cue use [(19), p. 11], suggesting that independent engagement with therapy is user-dependent and not always effective.

Therefore, the current pilot study aimed (1) to observe the behaviors of people independently engaging with a tablet-based application and (2) to pilot a protocol that taught individuals ways to enhance lexical retrieval attempts in both home and clinical environments of therapy. To accomplish this, we investigated the effect of a 10-week lexical retrieval protocol on participant behaviors of response latency, proportion of cue use, independent engagement with the treatment, and task accuracy. The protocol examined in the current work looks at ways to apply best research evidence into clinical practice that is supported by technology to promote enhanced outcomes. We hypothesized that lexical retrieval training could be used to teach PWA to increase autonomous effortful lexical retrieval attempts in tablet-based language rehabilitation targeting word finding. Successful adherence to the trained protocol was predicted to lead to a delayed response selection and a reduced cue use, as both of these behaviors were trained throughout treatment. Therefore, we predicted that individuals who received training would have a longer response and use a lower proportion of cue use and that these behaviors would not differ by location. As longer latencies and reduced cue use are likely to reflect the kind of independent and effortful lexico-semantic processing that is described in studies that examine errorful learning (2, 3, 22, 23), individuals who received training were hypothesized to have greater accuracy on treatment items and standardized assessments of naming.

## Materials and Methods

### Participants

The participants in this study included six adults with chronic post-stroke aphasia (four males and two females, mean age = 62.3, SD = 6.72) and were recruited through the MGH Institute of Health Professions Aphasia Center and *via* word of mouth. The Institutional Review Board (IRB) research consent form was reviewed with each participant and individuals provided informed consent prior to the initiation of the study. In order to be eligible, individuals had to have aphasia and be in the chronic stages of aphasia at least 6 months post-onset. Five of the participants had aphasia subsequent to left hemisphere strokes, whereas the aphasia of one participant (Trained 1) was related to a left hemisphere tumor resection. Participants had to be between the ages of 18 and 85 years and pre-morbidly right-handed English speakers with no history of significant psychiatric or medical disease. Participants also had to demonstrate impairments in naming ability as demonstrated on the Boston Naming Test (BNT) (48), the Naming and Word Finding subtests of the Revised Western Aphasia Battery (WAB-R) (49), and patient interview (see Table 1).

**Table 1:** Profiles of the patients enrolled in the 10-week protocol.

**Participant**	**Age (years)**	**Chronicity of stroke (years)**	**Aphasia diagnosis**	**Memory^a^**	**Attention^a^**	**Executive function^a^**	**Visuospatial ability^a^**	**WAB-R AQ: pre**	**WAB-R AQ: post**	**WAB-R NWF^b^: pre**	**WAB-R NWF^b^: post**	**BNT: pre**	**BNT: post**
Trained 1	64.9	9.5	Anomic	152, mild	140, mild	8, severe	53, mild	75.4	79.1	60	80	33	41
Trained 2	69.7	3.5	Anomic	143, moderate	156, mild	20, mild	79, mild	77.8	86	63	81	26	39
Trained 3	68.8	11.7	Anomic	191, mild	205, WNL	26, WNL	95, WNL	69.9	76.7	56	66	20	32
Untrained 1	52.3	8.06	Broca's	60, severe	185, WNL	24, WNL	102, WNL	33.8	41.8	31	31	19	NA
Untrained 2	59.9	29.0	TCM	123, moderate	184, WNL	24, moderate	92, WNL	67.7	68.2	92	95	51	55
Untrained 3	58.3	5.1	Anomic	125, moderate	187, WNL	28, WNL	92, WNL	89.5	89.2	64	65	16	15

*WAB-R, Western Aphasia Battery - Revised; AQ, Aphasia Quotient; BNT, Boston Naming Test; WNL, within normal limits*.

*All columns marked with “a” indicate that these are subtests of the Cognitive Linguistic Quick Test (50). All columns marked with “b” indicate that they are derived from the WAB-R*.

In order to be eligible, participants had to achieve a minimum score of 70% on the auditory comprehension subtests of the WAB-R (including yes/no questions, auditory word recognition, and sequential commands), as multiple therapy tasks required attending to spoken instructions and/or spoken stimulus items. The presence of a field cut as determined by the Cognitive and Linguistic Quick Test (50) symbol cancellation task would render participants ineligible as this could interfere with the ability to look at all portions of the iPad screen. As part of their first intervention session, the participants were taught to log in to the therapy application, make button responses, and turn the iPad on and off. Participants were provided with a printed handbook of instructions on these tasks and with contact information of the research team to help with any technical difficulties. This informal instruction period also served as a screen to ensure that the participants could meet the study participation demands of logging into the application and making button presses on the touch screen. In a follow-up to this pilot study, our lab has developed an iPad navigation screening and teaching tool to more formally evaluate baseline abilities to perform tasks on an iPad and/or learn to perform tasks on an iPad (51).

### Procedure

All the participants were involved in a 10-week treatment study that used a tablet-based language therapy application, Constant Therapy, a research-based language rehabilitation program devised by researchers at Boston University that incorporates tasks to address many domains of language and has been used in research studies investigating aphasia rehabilitation (Constant Therapy, Inc., Newton, MA, USA) (14, 15, 44, 52). Constant Therapy was selected because it allows for the tracking of response latency, cue use, and response accuracy, measures that enabled us to evaluate behaviors both in the clinic and during home practice. Participants were pseudo-randomly assigned to the Untrained group, where they would independently engage with therapy, or the Trained group, where they would receive training targeting effortful lexical retrieval. The three participants in the Untrained group had diagnostic profiles of anomic, Broca's, and transcortical motor aphasia. Of the three participants assigned to the Trained group, all had diagnoses of anomic aphasia. While the distribution of diagnoses across groups was initially more balanced, two additional participants initially enrolled in the study discontinued their participation shortly after consent.

### Pre- and Posttreatment Assessments

All the participants completed standardized pre- and posttreatment assessments to measure cognitive and linguistic ability, the 60-item BNT (48), the WAB-R [WAB; (49)], and the CLQT (50). Posttreatment assessments were administered by study staff who were blinded to group assignment. Due to scheduling conflicts, this was not the case for participant Trained 1, whose posttreatment assessment had to be completed by the final author and did not include the BNT.

### Experimental Tasks

The experimental tasks used in our treatment protocol were selected from the tablet-based Constant Therapy treatment application (4, 16) based on their effectiveness in targeting anomia and the limited task demands on reading. Furthermore, the selected tasks fell into one of two categories of either requiring or not requiring the covert or silent retrieval of a target word to successfully complete a trial. The tasks utilized in this study were category matching (CM), feature matching (FM), rhyming (RH), and syllable identification (SI). For each analyzed task, the picture of a noun appeared on screen accompanied by a spoken task prompt, written feature, or category, and participants made a response *via* screen touch. Nouns represented a wide variety of semantic categories, including but not limited to animals, furniture, body parts, and clothing. CM and FM engaged participants in considering the category membership of an item or the semantic features associated with a pictured item. In CM, the participants were instructed to select the correct category from a choice of three. In FM, the participants were instructed to press “Yes” or “No” to indicate whether an item had a feature or not (e.g., pictured item: banana, feature “has legs”). During the CM and FM tasks, lexical–semantic representations are thought to be improved through a strengthening of the feature and category associations with the target (53–56). Retrieval of the target word form was not necessary to perform the tasks.

In contrast, the RH and SI tasks required the covert retrieval of the exact word form of a pictured target to make an informed response for each trial. In RH, the participants were asked to indicate whether the name of a pictured target item rhymed with a spoken target item (e.g., pictured item: pen, spoken target “Does this rhyme with hen?”). For SI, the participants were asked to indicate whether the name of a pictured item had two syllables. For both the RH and SI, the participants indicated their response by selecting “Yes” or “No.” Participants could also press a button to hear the task prompt repeated.

For all tasks, a small audio icon in the upper corner of the picture target offered the opportunity to hear the name of the pictured item. We refer to this option as the *cue*, and participants in the Trained group were encouraged to refrain from using this cue button that provided the target word form until they had attempted to retrieve the target name independently (see details of training below). We anticipated that participants would show a reduced tendency to use the cue button on the CM and FM tasks, where the word form was not necessary to complete the task. In contrast, prior work with PWA completing the RH and SI tasks suggested that participants might exhibit a tendency for immediate and frequent use of the cue button to hear the target word since retrieving its word form was necessary to complete a trial. For all task trials, the participants received feedback related to their response accuracy in the form of a green check or a red “X” accompanied by a chime or a discrete buzz before proceeding to the next item. The application was programmed to present 15 trials of each task before moving on to the next. In the version of Constant Therapy available at the time of the study, item selection and presentation schedule (repeated vs. unique items) cannot be controlled by the clinician; therefore, the participants saw a mix of unique and repeated items.

### Trained vs. Untrained Therapy

Each participant was provided with an iPad that had access to Constant Therapy. All the participants attended 2 h of in-house therapy sessions at the MGH Institute of Health Professions Aphasia Center. Although a standardized assessment of reliability of treatment administration was not computed, each session was observed by either the first or the last author to assess the accuracy of protocol administration (on which feedback was provided following the session). Additionally, all the participants were encouraged to independently complete the assigned therapy program tasks once a day from home. At the start of each clinic session, clinicians reviewed the login frequency with each participant based on the following protocol:

1. I see that you logged in *X* times since I last saw you.
  - If logins are daily: I'm glad to see that you're using the app frequently.
  - If logins are infrequent: I see that you didn't log in very much. What happened?
2. Did you have any trouble using the iPad or logging in to Constant Therapy?
3. Is there any task that you feel is particularly difficult?

Afterwards, clinicians asked the participants to log in to Constant Therapy to complete their task battery. Participants in the Untrained group, as the name suggests, completed all of the Constant Therapy tasks independently. During the clinic sessions, clinicians scored the performance and observed how patients naturally interacted with the therapy application. Clinicians were allowed to provide simple clarification of task instructions and had scripts that provided them with acceptable ways to review the definition of a rhyme and a syllable (see Supplementary Materials for the task-specific protocol instructions that the Trained group received). Beyond instruction and keyword clarifications, clinicians were not allowed to provide additional semantic information, cues, or response guidance. If the Untrained participants asked questions or solicited additional feedback, clinicians were instructed to encourage participants to “make your best response” and to complete the tasks independently.

For the Trained group, the focus of in-clinic sessions was to (a) encourage lexical retrieval attempts on every trial of each task and (b) teach participants to review responses after receiving incorrect feedback. After clinicians assessed the login frequency of each participant, the participants were asked to log in to the Constant Therapy application. Then, clinicians instructed the participants to: “try to name every item that you see” on every task trial. Clinicians reminded the participants that the cue button, when pressed, would state the pictured item name. Participants were informed that they should *not* press the cue button until they had tried to retrieve the name and responded to the trial. Following this instruction, if the participants made attempts to select the cue button, the clinicians would stop the participant, stating, “Wait, I want you to think of the name first. Make your best guess and listen to the name after.” If participants selected the cue, clinicians prompted them to repeat the name after listening to it. Anytime feedback from the application indicated that a response was incorrect, the participants were instructed to pause, reflect upon their answer, and review the correct response before moving on to the next trial. Furthermore, clinicians encouraged participants to use these strategies when practicing tasks independently at home (see Supplementary Materials).

This overt lexical retrieval protocol was based on the principle that effortful lexical retrieval attempts and independent engagement with therapy can improve naming (3, 22). The protocol was also designed to include components that could be reliably measured and tracked throughout the course of therapy both in-house and during independent home practice in a realistic manner for clinicians: response latency, cue use before response selection, trials completed per login, and accuracy. Furthermore, this protocol was applied to tasks that either did or did not require the overt verbalization of a lexical item. Adherence to the protocol was expected to lead to increased response latencies and a reduced proportion of cues, and that these behaviors would carry over to home practice. The metrics of latency and cue use were automatically tracked by Constant Therapy and therefore provided a means of inferring protocol application during home-based logins.

### Analysis

The Constant Therapy application collected and tracked data and thus generated reports that included measures of login times, response selection accuracy, response latency time, number of cue requests, and latency before cue selection. Based on the dates listed in the output, we were able to calculate the total number of days the participants logged into treatment at home. Data from a total of 36,464 trials was accrued over the duration of the study. Trials with response latencies 3 standard deviations above the mean (by participant) were excluded from analyses as these were unrepresentative of overall behavior and indicated an interruption to therapy (as the application did not time out on its own if a participant ended a login mid-trial). Of the remaining 34,688 trials, we measured the intensity of treatment by calculating the total number of unique login (averaging across home and clinical practice), as well as the average number of trials per login per week, for each participant. Then, we calculated the following measures by unique participant login by task: (1) the average latency (in seconds) before a response was selected in a trial; (2) the average proportion of cues (playing audio recordings of the pictured item names) selected before a response per trial; (3) the number of trials completed; and (4) the average accuracy.

We used linear mixed-effects models for our regression analyses to estimate the extent to which factors of group (Trained vs. Untrained), location (clinic vs. home), and time (days) explained the outcomes on measures of response latency, cue use before response selection, trials per login (intensity), and accuracy measured throughout the course of treatment [e.g., utilizing the linear mixed-effects model (fixed = Measure ~ Group^*^Location^*^Time, reStruct = (1|Participant), data = datafile, method = “REML”] (57). In our model, the participant variable was designated as the random effects. Since the requirements for lexical retrieval differed by task type, data for the CM and FM tasks (CM+FM) and the RH and SI tasks (RH+SI) were respectively grouped together in analyses. Figures for behaviors of latency and cue use separated by each task are available in Appendices B, C. We analyzed our measures of interest within and across groups and therapy contexts in order to investigate the impact of the training protocol and whether this protocol would carry over to home practice. We used Tukey's *post-hoc* tests to further interpret significant interaction effects from our linear mixed-effects regressions. We also calculated the number of unique logins each participant completed.

To probe for generalized improvement, we report on changes between the pre- and post-assessment scores and use the benchmarks proposed in Gilmore et al. (58) for the WAB-R Aphasia Quotient (AQ) and BNT. Additionally, to account for the heterogeneity of the baseline scores within and between groups, we examined item-level improvement on the WAB-R (Aphasia Quotient composite score, Naming and Word Finding subtest) and BNT in every individual participant by computing Marx and Cummings' (59) normalized change scores. Normalized change (*c*) scores differ from change scores (post – pre) as they allow us to determine the overall level of improvement someone demonstrates on a measure relative to their baseline performance and the maximum possible change in score. Following the protocol detailed in Marx and Cummings (59), *c* scores were calculated as POST – PRE/MAXIMUM POSSIBLE SCORE – PRE; if a participant demonstrated a loss, the calculation was POST – PRE/PRE, and if there was no change, the score was 0. Finally, we calculated the percentage of treatment items that each participant saw that overlapped with items on the WAB-R and the BNT. Analyses were conducted utilizing R version 1.2.1335. Mixed-effects regressions were conducted utilizing lme4 (57) and plots were generated using ggplot2 (60).

## Results

Behavioral means and standard errors of response latency, proportion cue use, trials per login, and accuracy were computed for all task types and are reported in Table 2 for reference. Participant-specific means are reported in Appendix A. Means are reported for the first week of participation (excluding the first clinic session as the participants were unfamiliar with the therapy tasks and protocol) and the final week of intervention to reflect patterns of behaviors by group in the initial and final stages of treatment.

**Table 2:** Mean latency, cue use, trials, and accuracy for CM+FM and RH+SI for both the Untrained and Trained groups by time point.

**Task**	**Group**	**Location**	**Week**	**Latency**	**SE**	**Cue use**	**SE**	**Trials/Login**	**SE**	**ACC**	**SE**
CM+FM	Trained	Clinic	1	15.04	6.22	0.30	0.30	27.8	10.8	0.79	0.06
			10	16.12	8.59	0.00	0.01	19.0	5.43	0.90	0.03
		Home	1	9.97	3.15	0.15	0.18	41.7	16.5	0.79	0.07
			10	10.83	1.19	0.00	0.00	20.0	0.00	0.88	0.03
	Untrained	Clinic	1	12.02	5.34	0.48	0.28	47.9	24.9	0.81	0.11
			8	9.04	4.10	0.27	0.16	40.0	15.9	0.82	0.02
		Home	1	7.22	1.53	0.26	0.22	24.8	8.15	0.77	0.10
			8	4.26	0.62	0.03	0.04	12.3	3.18	0.68	0.02
RH+SI	Trained	Clinic	1	18.33	12.73	0.87	0.60	27.1	12.9	0.81	0.05
			10	7.10	0.16	0.68	0.23	30.0	0.00	0.47	0.03
		Home	1	18.1	18.49	0.59	0.54	41.1	16.7	0.69	0.04
			10	17.7	5.79	0.20	0.20	21.8	3.40	0.68	0.03
	Untrained	Clinic	1	11.9	2.89	0.94	0.43	48.1	25.3	0.73	0.08
			10	9.77	1.68	1.39	0.13	27.0	14.0	0.75	0.03
		Home	1	10.9	5.08	0.93	0.43	24.8	8.4	0.63	0.01
			10	6.79	0.98	0.99	0.32	15.0	0.73	0.68	0.02

*CM+FM, category matching + feature matching; RH+SI, rhyming + syllable identification; ACC, accuracy*.

### Latency

The linear mixed-effects regression results demonstrate a main effect of group, with the Trained group showing longer response times on CM+FM (*p* = 0.004) and RH+SI (*p* = 0.002) task types (see Table 3). There were no main effects of location or time. The interaction of group and time was significant for both CM+FM (*p* = 0.008) and RH+SI (*p* = 0.009).

**Table 3:** Results from the mixed-effects linear model analyses examining the relationship of group (factor; trained vs. untrained), location (factor; clinic vs. home), and *p < 0.05, **p < 0.01, ***p < 0.001, ****p < 0.0001.

**Measure**	**Task**	**Parameter**	**Estimate**	**95% CI: Upper**	**95% CI: Lower**	**SE**	***t***	***p***
Latency	CM+FM	(Intercept)	16.2	20.4	12.0	2.12	7.65	<0.0001
		Untrained	−8.97	−2.97	−14.9	3.06	−2.93	0.004**
		Home	−3.75	1.07	−8.57	2.46	−1.53	0.128
		Time	−0.04	0.06	−0.14	0.05	−0.94	0.347
		Untrained × Home	2.09	9.36	−5.18	3.71	0.56	0.574
		Untrained × Time	0.21	0.37	0.05	0.08	2.65	0.008**
		Home × Time	0.12	0.22	0.02	0.05	2.41	0.017*
		Untrained × Home × Time	−0.25	−0.07	−0.43	0.09	−2.88	0.004**
	RH+SI	(Intercept)	39.3	54.3	24.3	7.67	5.13	<0.001
		Untrained	−39.7	−15.4	−64.0	12.4	−3.20	0.002**
		Home	12.2	29.4	−5.05	8.80	1.38	0.168
		Time	−0.28	0.03	−0.59	0.16	−1.76	0.079
		Untrained × Home	−1.83	25.4	−29.1	13.9	−0.13	0.896
		Untrained × Time	0.67	1.16	0.18	0.25	2.63	0.009**
		Home × Time	−0.19	0.16	−0.54	0.18	−1.06	0.289
		Untrained × Home × Time	−0.17	0.38	−0.72	0.28	−0.62	0.534
Cue use	CM+FM	(Intercept)	0.23	0.31	0.15	0.04	5.08	<0.001
		Untrained	0.17	0.29	0.05	0.06	2.64	0.009**
		Home	−0.09	0.01	−0.19	0.05	−1.74	0.083
		Time	0.00	0.00	0.00	0.00	−2.54	0.011*
		Untrained × Home	−0.11	0.03	−0.25	0.07	−1.34	0.180
		Untrained × Time	0.00	0.00	0.00	0.00	−1.00	0.318
		Home × Time	0.00	0.00	0.00	0.00	1.37	0.173
		Untrained × Home × Time	0.00	0.00	0.00	0.00	0.41	0.680
	RH+SI	(Intercept)	0.00	0.43	−0.43	0.22	−0.01	0.989
		Untrained	1.79	2.49	1.08	0.36	4.95	<0.001****
		Home	0.44	0.95	−0.07	0.26	1.71	0.089
		Time	0.01	4.62	−4.59	0.00	2.35	0.019*
		Untrained × Home	−0.46	0.34	−1.26	0.41	−1.11	0.266
		Untrained × Time	−0.03	−0.01	−0.05	0.01	−3.34	<0.001***
		Home × Time	−0.01	0.01	−0.03	0.01	−2.36	0.019*
		Untrained × Home × Time	0.02	0.04	0.00	0.01	1.97	0.049*
Trials/Login	CM+FM	(Intercept)	43.5	51.6	35.3	4.16	10.5	<0.001
		Untrained	−0.81	10.9	−12.6	5.99	−0.13	0.893
		Home	4.44	13.9	−5.03	4.83	0.92	0.359
		Time	−0.08	0.09	−0.26	0.09	−0.89	0.375
		Untrained × Home	−25.0	−10.7	−39.3	7.28	−3.44	<0.001***
		Untrained × Time	−0.01	0.28	−0.30	0.15	−0.09	0.931
		Home × Time	−0.15	0.05	−0.35	0.10	−1.54	0.124
		Untrained × Home × Time	0.17			0.17	0.97	0.333
	RH+SI	(Intercept)	40.1	48.5	31.7	4.29	9.35	<0.001
		Untrained	1.58	13.5	−10.4	6.09	0.26	0.796
		Home	5.25	15.7	−5.16	5.31	0.99	0.323
		Time	−0.07	0.11	−0.25	0.09	−0.71	0.475
		Untrained × Home	−25.6	−10.6	−40.6	7.63	−3.36	<0.001***
		Untrained × Time	−0.06	0.23	−0.35	0.15	−0.38	0.706
		Home × Time	−0.18	0.06	−0.42	0.12	−1.55	0.122
		Untrained × Home × Time	0.25	0.60	−0.10	0.18	1.37	0.172
Accuracy	CM+FM	(Intercept)	0.78	0.84	0.72	0.03	578.6	<0.001
		Untrained	0.02	0.16	−0.12	0.07	0.06	0.814
		Home	0.02	0.08	−0.04	0.03	0.35	0.551
		Time	0.00	0.00	0.00	0.00	3.02	0.082
		Untrained × Home	−0.02	0.04	−0.08	0.03	0.49	0.485
		Untrained × Time	0.00	0.00	0.00	0.00	0.30	0.583
		Home × Time	0.00	0.00	0.00	0.00	5.34	0.021*
		Untrained × Home × Time	0.00	0.00	0.00	0.00	4.23	0.040*
	RH+SI	(Intercept)	0.77	0.83	0.71	0.03	22.2	<0.001
		Untrained	−0.07	0.03	−0.17	0.05	−1.32	0.186
		Home	−0.07	0.01	−0.15	0.04	−1.52	0.129
		Time	0.00	0.00	0.00	0.00	−2.18	0.030*
		Untrained × Home	−0.06	0.06	−0.18	0.06	−1.02	0.306
		Untrained × Time	0.00	0.00	0.00	0.00	2.13	0.034*
		Home × Time	0.00	0.00	0.00	0.00	1.41	0.159
		Untrained × Home × Time	0.00	0.00	0.00	0.00	−0.69	0.493

*CM+FM, category matching + feature matching; RH+SI, rhyming + syllable identification. Time (continuous; days)*.

**Figure 1:**
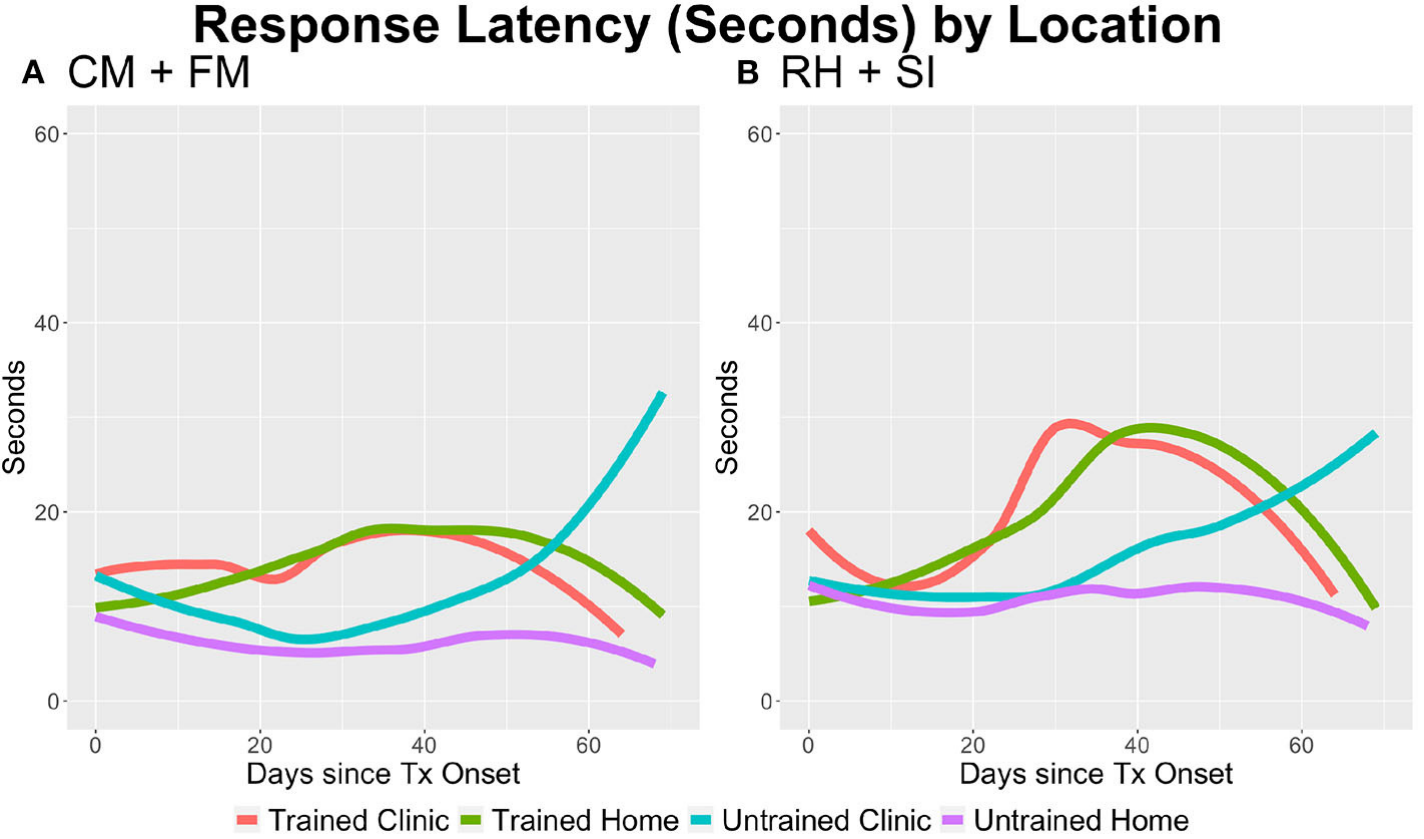
The average response latency per trial by task type, group, and location. **(A)** On category matching + feature matching (CM+FM), the Trained group took longer per trial (*p* = 0.002) and over time in the home setting (*p* = 0.004). The Untrained group took longer over time (*p* = 0.008), and all participants took longer in the home setting (*p* = 0.017). **(B)** On rhyming + syllable identification (RH+SI), the Trained group took longer per trial (*p* = 0.002) and the Untrained group took longer over time (*p* = 0.009).

For CM+FM, additional interactions of location and time (*p* = 0.017), where the response latencies were longer at home, and group, location, and time (*p* = 0.004) were significant (see Figure 1 and Tables 2, **3**). Tukey's *post-hoc* tests demonstrated that on CM+FM in clinic, the Trained group took longer than the Untrained group on days 1–49 at *p* < 0.05. Beginning on day 63, the Untrained group took significantly longer on the CM+FM treatment task trials than did the Trained group in clinic (*p* < 0.001). The Trained group took significantly longer at home through treatment between all days 1–70 at *p* < 0.05. On RH+SI, the Trained group took significantly longer in clinic between days 1–2 and 8–49. Between days 63 and 65, the Untrained group took significantly longer on trials in clinic at *p* < 0.01. The Trained group took significantly longer on the RH+SI task trials at home at *p* < 0.05 between days 8 and 70.

### Cue Use Before Response Selection

**Figure 2:**
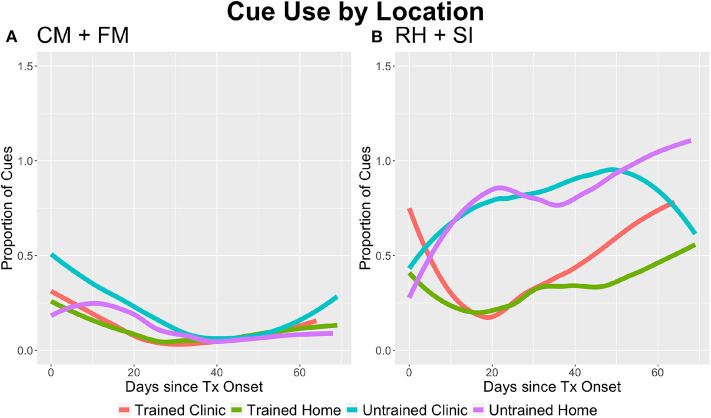
The average proportion of cues used per trial by task type, group, and location. **(A)** On category matching + feature matching (CM+FM), the Trained group used a lower proportion of cues per trial (*p* = 0.009) and all groups used less cues over time (*p* = 0.011). **(B)** For rhyming + syllable identification (RH+SI), the Trained group used less cues than the Untrained group (*p* < 0.001); however, the Untrained group used less cues over time (*p* < 0.001), but more cues than the Trained group over time during independent practice at home (*p* = 0.049). For all participants, cue use increased overall over time (*p* = 0.019), but decreased in the home setting (*p* = 0.019) as treatment progressed, suggesting that cue use in clinic increased.

The main effect of group was significant for cue use on CM+FM (*p* = 0.009) and RH+SI (*p* < 0.0001; see Figure 2), where the Untrained group used a higher proportion of cues per trial than did the Trained group. There was no significant main effect of location for either task type. The main effect of time was significant for CM+FM (*p* = 0.011), where cue use went down over time for all groups. While the main effect of time was also significant for RH+SI, the reverse happened and cue use increased over time (*p* = 0.019). There were no significant interactions of group, location, and time for CM+FM. For RH+SI, there were significant interactions of location and time (*p* = 0.19), group and time (*p* < 0.001), and group, location, and time (*p* = 0.049; see Figure 2). Tukey's *post-hoc* tests revealed that the Untrained group used significantly more cues than did the Trained group on CM+FM at *p* < 0.05 in clinic between days 12 and 41 and at home between days 7 and 55. On RH+SI, the Trained group used significantly fewer cues at *p* < 0.05 between days 8 and 59 in clinic and between days 2 and 70 at home.

### Trials per Login (Intensity) and Logins

**Figure 3:**
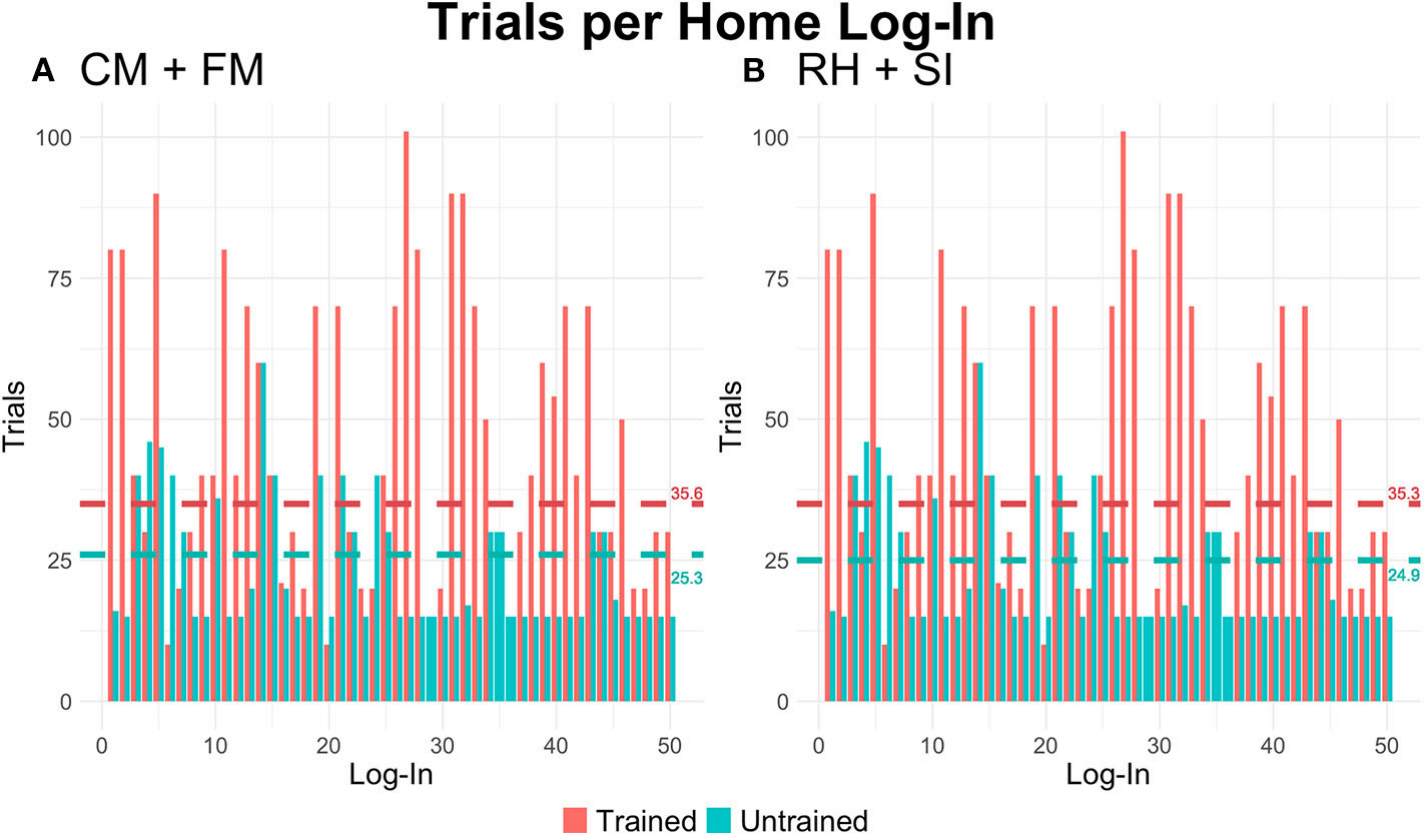
The average number of trials per unique login by task type and group during independent home practice. *Horizontal lines* represent the average number of trials completed per login by group (*teal*: Untrained). **(A)** For category matching + feature matching (CM+FM), the Untrained group completed significantly fewer trials at home than the Trained group (*p* < 0.001). **(B)** Consistent with behaviors reported for CM+FM, Untrained participants completed significantly fewer trials during independent practice at home on the rhyming + syllable identification (RH+SI) tasks (*p* < 0.001).

There were no significant main effects of group, location, or time for CM+FM or RH+SI. The interaction of group and location was significant for both CM+FM (*p* < 0.001) and RH+SI (*p* < 0.001), where the Trained group completed significantly more trials per login at home than did the Untrained group (see Figure 3). By the end of treatment, the Untrained group completed an average of 37 unique logins (SE = 6.37) and the Trained group averaged 50 (SE = 9.42), as averaged across locations.

### Accuracy

**Figure 4:**
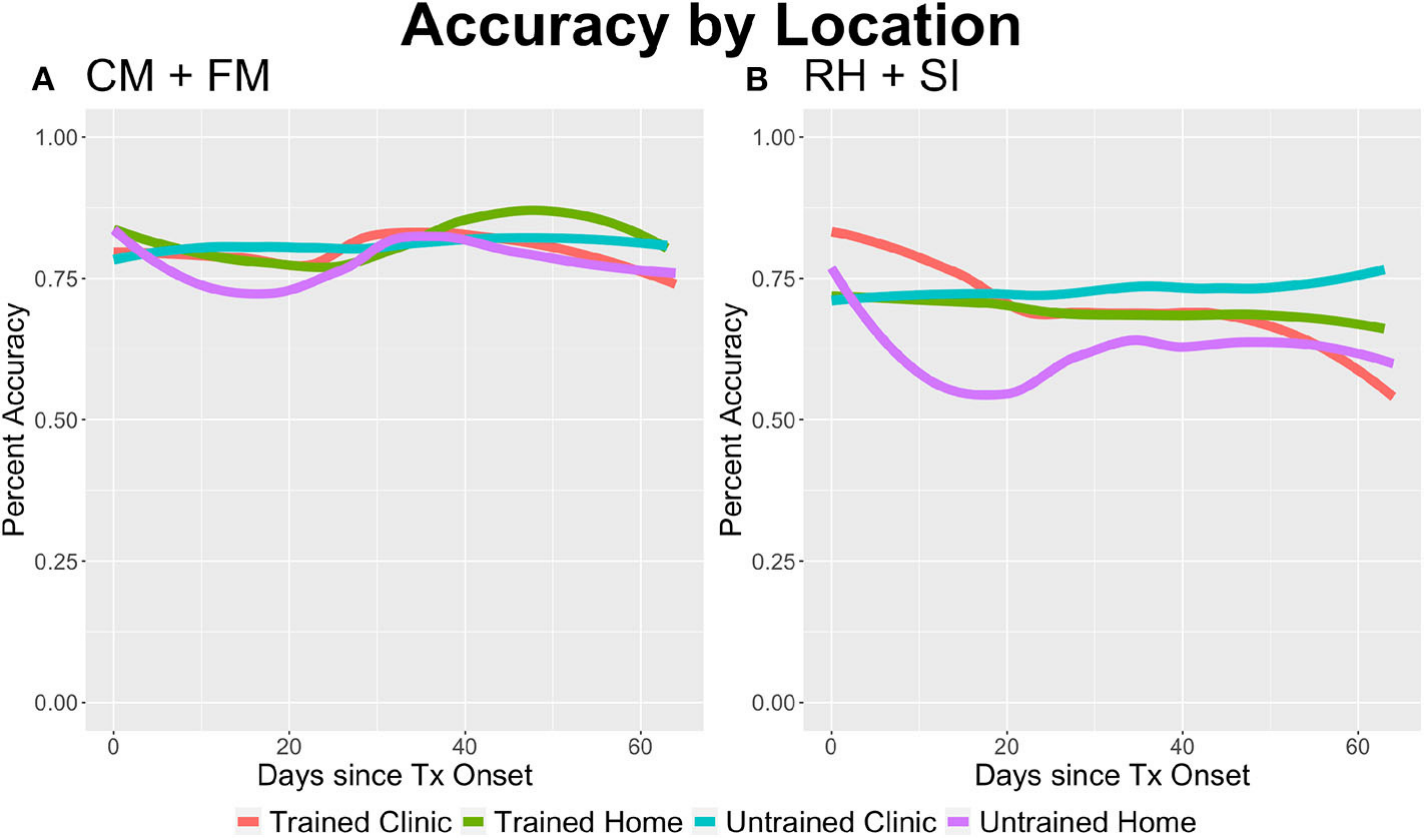
The average accuracy on trials by task type, group, and location. **(A)** All participants achieved higher accuracies during independent practice at home on category matching + feature matching (CM+FM) (*p* = 0.021); additionally, the Untrained group achieved higher accuracies at home over time (*p* = 0.040). **(B)** All participants improved their performance over time on rhyming + syllable identification (RH+SI) (*p* = 0.030), where the Untrained group made greater gains on treatment items over time (*p* = 0.034).

While there were no main effects of group, location, or time for CM+FM, for RH+SI, there was a significant main effect of time (*p* = 0.030), with accuracy decreasing over time, the effect driven by clinic performance for the Trained group and home performance for the Untrained group (see Figure 4). The interaction of location and time (*p* = 0.021) was significant for CM+FM, with home accuracy being lower than clinic accuracy in the early third of therapy and becoming more similar as therapy progressed. The interaction of group, location, and time (*p* = 0.04) was also significant for CM+FM, where the Trained group performed similarly in clinic and at home in the early phases of therapy, where scores in the final third of therapy were higher at home than in clinic. For the Untrained group, accuracy was lower at home than in clinic in the early phases of therapy and showed the opposite pattern late in therapy. Furthermore, Tukey's *post-hoc* testing demonstrated that the Trained group performed higher at home than did the Untrained group between days 1 and 56 on CM+FM at *p* < 0.05. For RH+SI, the interaction of group and time was significant (*p* = 0.034), where Tukey's *post-hoc* testing demonstrated that the Untrained group performed significantly higher than the Trained group in clinic at *p* < 0.05 between days 21 and 70, but significantly lower than the Trained group at home at *p* < 0.05 between days 8 and 69.

### Standardized Assessment Outcomes

**Figure 5:**
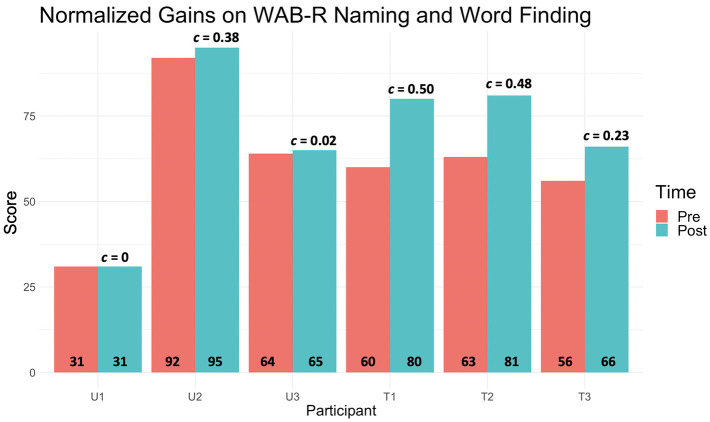
The pre- (*orange*) and post- (*teal*) WAB-R Aphasia Quotient composite scores for each participant, where the normalized gain (*G*) scores are placed *above the corresponding bars* and raw pre- and post-scores *at the bottom of each*. All Trained participants' gain scores were close to medium effect posttreatment, whereas only one Untrained participant achieved the same.

Two of the Trained participants and one Untrained participant met the Gilmore et al. (58) 5.03 benchmark of significant change on the WAB-R AQ. Similarly, all three Trained participants but only one Untrained participant (Untrained 2) met the 3.30 benchmark of significant change on the BNT. All three Trained participants achieved normalized gains with small to medium effects (one medium) on the WAB-R AQ, WAB-R Naming and Word Finding subtest (two medium), and BNT (three medium). In the Untrained group, one participant achieved normalized gain with small effect on the WAB-R AQ, whereas another made gains with medium effect on both the WAB-R Naming and Word Finding subtest and the BNT (see Table 4 and Figure 5). We also calculated the percentage of items that participants saw throughout the course of treatment that were also on the WAB-R and BNT. For CM+FM, 2.37–4.06% (mean = 3.02, SE = 0.01) of the items the Trained participants saw were on the WAB-R and 5.99–7.53% were on the BNT (mean = 6.39, SE = 0.22). Similarly, for RH+SI, 1.52–2.37% (mean = 2.01, SE = 0.13) of the treatment items overlapped with items on the WAB-R and 5.32–7.29% on the BNT (mean = 5.40, SE = 0.2) for the Trained participants. For the Untrained participants, 1.88–2.71% of the treatment items overlapped with items on the WAB-R (mean = 2.49, SE = 0.20) and 5.27–8.78% on the BNT (mean = 6.38, SE = 0.31) on CM+FM. Finally, on RH+SI, 1.53–1.63% (mean = 1.56, SE = 0.20) of the items the Untrained participants saw overlapped with items on the WAB-R and 4.56–5.58% (mean = 5.26, SE = 0.28) on the BNT.

**Table 4:** Change scores and Marx and Cummings' (2007) normalized gain scores on standardized assessments of language.

**Assessment**	**Group**		**Pre-treatment scores**	**Post-treatment scores**	**Change scores**	***c* scores**
WAB-R AQ	Trained	1	75.4	79.1	3.70	0.15
		2	77.8	86	8.20	0.38
		3	69.9	76.7	6.80	0.23
	Untrained	1	33.8	41.8	8.00	0.12
		2	67.7	68.2	0.50	0.02
		3	89.5	89.2	−0.30	−0.03
WAB-R NWF	Trained	1	60.0	80.0	20.0	0.50
		2	63.0	81.0	18.0	0.49
		3	56.0	66.0	10.0	0.23
	Untrained	1	31.0	31.0	0.00	0.00
		2	92.0	95.0	3.00	0.37
		3	64.0	65.0	1.00	0.03
BNT	Trained	1	33.0	41.0	8.00	0.30
		2	36.0	39.0	13.0	0.38
		3	20.0	32.0	12.00	0.30
	Untrained	1	19.0	NA	NA	NA
		2	51.0	55.0	4.00	0.44
		3	16.0	15.0	−1.00	−0.02

*For the interpretation of results, c scores below 0.30 are considered to have a low effect, scores between 0.30 and 0.70 medium effect, and scores above 0.70 a large effect (59, 61). WAB-R, Revised Western Aphasia Battery; BNT, Boston Naming Test*.

## Discussion

The current manuscript reports on a pilot study that aimed to examine and characterize behaviors of latency and cue use in individuals with aphasia engaged in tablet-based treatment for anomia. Tablet-based interventions are increasingly being utilized in aphasia rehabilitation with the goal of increasing patient access to therapy. Research is beginning to demonstrate the efficacy of tablet-based applications (4, 5, 8, 16). In the current work, our approach aimed to explore the untrained behaviors used by PWA while engaging in tablet-based anomia intervention and to evaluate these relative to the behaviors of PWA trained to delay response selection until independent lexical retrieval was attempted with a limited reliance on cues.

Therefore, the goals of this study were: firstly, to characterize PWA's behaviors during tablet-based treatment during independent completion of tasks; secondly, to see whether optimal behaviors (independent retrieval of lexical items without a reliance on cues) could be taught with strategy training; and thirdly, to see whether PWA would carry over the use of strategies at home without clinician presence and encouragement. Prior clinical experience with tablet-based intervention suggested that many PWA utilize cues provided by the application with little evidence of initiating lexical retrieval attempts independently (PWA have been observed to immediately request cues that verbalize the target item name and use this to inform their response), with little evidence of pausing on every trial and/or verbalizing item names. Therefore, the major focus of the protocol training was to direct individuals to attempt to retrieve a target lexical item before requesting cues integrated in the app, thereby applying principles intended to enhance gains during errorful intervention (3, 22, 26). Evaluating measures of response latency and the proportion of cues used tracked by the application allowed for an investigation of behaviors in both clinic and home settings accrued across the completion of many trials. Although the current sample size is small, the study was conducted within a realistic therapy context relevant to the current care process with intensive tracking of every individual trial each participant completed.

Our results suggest that training strategies to independently retrieve lexical targets and to acknowledge and review incorrect responses can alter and potentially improve PWA's engagement with teletherapy. We first examined the response latency and cue use, behaviors that were targeted by the training protocol. Across both task types, the Trained group took longer and used less cues than did the Untrained group. Furthermore, while there was an interaction of time and location for the Untrained group, the Trained group response latencies did not differ significantly by location. As predicted, cue use overall was lower for the CM+FM than the RH+SI tasks, where the former task type did not require the retrieval of word form to complete the therapy task. Even so, behaviors of latency and cue use differed between the Trained and Untrained group, suggesting that the strategy training influenced behaviors for tasks that did and did not require retrieval of the target word form. Strategy training in aphasia rehabilitation has primarily focused on teaching communicative partners strategies to support communication (62–64) and the training of augmentative or compensatory strategies to individuals with aphasia to assist the success and management of conversation (65–67). The preliminary results from our protocol suggest that individuals with aphasia are capable of learning strategies that aim to enhance the restoration of lexical retrieval and that the addition of these strategies to therapy targeting anomia may lead to greater naming gains. Furthermore, the preliminary finding that the application of strategy training led to greater generalized gains following restorative therapy motivates a reexamination of what providing restorative therapy, and ensuring the successful restoration of impaired or lost function, truly entails in the context of clinical practice, whether in person or *via* teletherapy.

We then examined the intensity of treatment as a measure of the number of trials completed per treatment login. For all tasks, the Trained group engaged more frequently (number of logins) and, furthermore, more intensely at home (number of trials per login) than did the Untrained group.

As all participants were encouraged to independently engage with the application as much as possible from home, a possible explanation is that the increased engagement of the clinician during the in-house clinic sessions for the Trained group relative to the Untrained group led to an increased motivation or attention of participants to continue to practice independently, though we acknowledge that other factors could also account for this difference. Prior research has shown that active engagement or strategy training can influence motivation and learning during rehabilitation (68–71). Skidmore et al. (71), for example, examined apathy, defined as the lack of motivation and interest, in 30 individuals from an in-patient stroke rehabilitation unit. All participants worked on four to six individualized rehabilitation goals throughout the study period, where 15 participants received additional strategy training to learn to self-evaluate and address goals through goal-setting, planning, and monitoring of performance. The researchers found that the strategy-trained group had lower scores of apathy and proposed that self-monitoring and problem solving may have promoted participant perseverance and engagement. Metacognitive training similarly teaches self-awareness and problem solving and has been thought to promote learning and motivation (68). Given these findings, the results of the current study are likely due to an increased monitoring of behaviors and self-evaluation that arose through the strategy training.

The average treatment task accuracy ranged from 67.2 to 78.7%, and accuracy on RH+SI actually decreased over time, driven by the Trained group's clinic performance. While cue use can assist a person to reach a correct response, the results suggest that task accuracy may not be the most important aspect of therapy. Prior work on retrieval practice has suggested that it is the combination of effortful retrieval and success that likely leads to the long-term benefits of learning conditions where the production of errors is not controlled (2, 3, 72). In conditions of learning where errors can occur, error detection or feedback is essential to support learning (26, 73–75). Constant Therapy tasks automatically offer feedback related to the accuracy of a response *via* visuals and audio, where the Trained group was additionally coached to press the cue button in the instance of negative feedback in order to hear the name of the target item and support the integration of learning. Importantly, as hypothesized, behaviors of increased latency and reduced cue use prior to response selection led to improved generalized outcomes.

Improvements on standardized assessment outcomes (WAB-R and BNT) were observed, where the Trained group more successfully met the benchmarks proposed in Gilmore et al. (58) and achieved higher normalized gains. The findings support prior work identifying superior outcomes in settings of effortful lexical retrieval (2, 3, 21, 22). The findings are also likely influenced by the increased number of logins and trials completed. One participant in the Untrained group (Untrained 2) improved on the Naming and Word Finding subtest of the WAB-R as well as the BNT. Interestingly, this participant self-developed strategies through the course of therapy. Of note is that this participant had a high naming ability as measured by the BNT and WAB-R at baseline, which may also explain why his accuracy on the treatment items did not change significantly over time. He was included in this study as he does present with anomia and describes this deficit as being a barrier in his communication. While his pretreatment performance was high, it is notable that he was still able to make gains, which may be attributable to the strategies he self-developed throughout the course of treatment. By the end of the study, by his own initiative, this participant wrote down any target for which he was uncertain of the response and documented the feedback provided by the app. This resulted in long delays before responding and drove many of the effects seen wherein the Untrained group showed increasing latencies of response in the final weeks of therapy (see Appendix A for individual participant means). The observation of this type of behavior is consistent with prior work examining cue use that determined that autonomous user engagement with therapy is variable (19). Some PWA may naturally develop strategies that support optimal engagement with tablet-based therapy applications, while others may benefit from training to better support their practice.

The findings provide encouraging pilot evidence to suggest that training can lead to increased lexical retrieval attempts and reduced cue use and, furthermore, that trained behaviors can be maintained from clinic to home practice. Furthermore, the intensive training of these behaviors appears to lead to increased autonomous engagement, as demonstrated by the increased intensity at which the Trained participants completed trials from home. The combination of increased response times, reduced cue use, and generalized treatment outcomes suggests that participants who spent more time per trial used this time to autonomously retrieve lexico-semantic information. If this change in cognitive process underlies the increased response times and reduced cue use, this theory, in addition to increased intensity, may explain why the Trained group demonstrated greater generalizable effects of treatment on standardized assessments of language, particularly the composite Aphasia Quotient of the WAB-R, and why the one individual of the Untrained group (Untrained 2) also improved. As such, as telepractice is increasingly utilized lieu of, or to support in-person treatment, it is essential to consider the role of the clinician in therapy and how clinic time can be spent training strategies that shape behaviors to promote outcomes or enhance engagement long term. Additional research will be needed to determine whether this is replicated and whether, as postulated, increased engagement of the clinician during clinic sessions promotes increased at-home practice.

### Limitations

The current study was a pilot study to examine behaviors throughout tablet-based treatment and how these relate to outcomes on task performance and standardized assessments. The study had limitations, which we acknowledge, and must be taken into consideration when interpreting the results. First, data were collected from a small and variable sample. Within this small sample, group assignment was pseudo-randomized, and unfortunately two participants dropped out shortly after being consented, meaning that the groups that were not equally matched on language or cognitive ability (see Table 1). We hope to have compensated for this issue by utilizing normalized gain scores and published benchmarks of significance to examine within-participant gains on standardized assessments in a more objective way.

We chose to use a therapy platform that is widely available on tablets in order to make findings relevant and realistic to real-world clinical practice. In the version of Constant Therapy used at the time of treatment, however, specific items and the frequency at which items are represented cannot be controlled for; therefore, we were unable to track item-level improvement based on intensity of practice, and this brings a reduction in experimental control. Future directions of this work will be to implement a protocol where item-level improvement can be systematically monitored to examine the treatment-specific effects of practice in a controlled single-subject design. Despite its limitations, we put forward this protocol training and pilot results to demonstrate a unique way to envision the role of the clinician when working with PWA and tablet-based applications in light of reduced hospitalization times and limited access to rehabilitative care.

## Data Availability Statement

The raw data supporting the conclusions of this article will be made available by the authors, without undue reservation.

## Ethics Statement

The studies involving human participants were reviewed and approved by The Mass General Brigham Institutional Review Board. The patients/participants provided their written informed consent to participate in this study.

## Author Contributions

RP, SP, and SV-R conducted all patient recruitment, experimental implementation, and gave substantial edits to the manuscript. SV-R is responsible for the experimental design. JG conducted all analyses and manuscript preparation. All authors contributed to the article and approved the submitted version.

## Conflict of Interest

The authors declare that the research was conducted in the absence of any commercial or financial relationships that could be construed as a potential conflict of interest.
