# Supplementary material for: The Application of Lexical Retrieval Training in Tablet-Based Speech-Language Intervention
Source: Front Neurol. 2020 Nov 16;11:583246. doi: 10.3389/fneur.2020.583246 (PMC7706007; doi:10.3389/fneur.2020.583246)
Supplement: Supplementary file 1 [file Data_Sheet_1.docx]

**APPENDIX A**

Average latency, cue use, trials per log-in, and accuracy by task-type, location, group, and week (1 and 10) for every individual participant who participated in the current study.

| Task | Group | Participant | Location | Week | Latency | SE | Cue Use | SE | Trials/Log-In | SE | ACC | SE |
| --- | --- | --- | --- | --- | --- | --- | --- | --- | --- | --- | --- | --- |
| CM+FM | Untrained | Untrained 1 | Clinic | 1 | 18.6 | 9.37 | 0.91 | 0.19 | 8.00 | 0.00 | 0.72 | 0.13 |
|  |  |  | Home | 1 | NA | NA | NA | NA | NA | NA | NA | NA |
|  |  |  | Clinic | 1 | 9.84 | 1.59 | 0.53 | 0.10 | 13.6 | 1.00 | 0.74 | 0.03 |
|  |  |  | Home | 10 | 6.72 | 0.26 | 0.27 | 0.04 | 14.1 | 0.33 | 0.75 | 0.02 |
|  |  | Untrained 2 | Clinic | 1 | 7.71 | 1.73 | 0.17 | 0.10 | 116 | 2.31 | 0.95 | 0.01 |
|  |  |  | Home | 1 | 9.40 | 1.66 | 0.06 | 0.06 | 28.0 | 6.93 | 0.96 | 0.02 |
|  |  |  | Clinic | 10 | 15.8 | 3.33 | 0.19 | 0.05 | 45.1 | 7.96 | 0.97 | 0.01 |
|  |  |  | Home | 10 | 11.9 | 2.92 | 0.08 | 0.04 | 29.9 | 2.43 | 0.97 | 0.01 |
|  |  | Untrained 3 | Clinic | 1 | 8.77 | 2.27 | 0.19 | 0.08 | 55.0 | 0.00 | 0.61 | 0.19 |
|  |  |  | Home | 1 | 6.12 | 2.05 | 0.00 | 0.00 | 15.0 | 0.00 | 0.60 | 0.27 |
|  |  |  | Clinic | 10 | 4.76 | 0.49 | 0.05 | 0.02 | 75.8 | 6.25 | 0.72 | 0.03 |
|  |  |  | Home | 10 | 4.11 | 0.17 | 0.02 | 0.01 | 21.7 | 1.54 | 0.74 | 0.02 |
|  | Trained | Trained 1 | Clinic | 1 | 16.7 | 5.61 | 0.03 | 0.03 | 43.0 | 7.00 | 0.80 | 0.06 |
|  |  |  | Home | 1 | 9.11 | 1.90 | 0.00 | 0.00 | 54.4 | 10.15 | 0.74 | 0.04 |
|  |  |  | Clinic | 10 | 11.9 | 1.15 | 0.07 | 0.01 | 52.1 | 4.64 | 0.74 | 0.02 |
|  |  |  | Home | 10 | 7.15 | 0.34 | 0.06 | 0.01 | 50.9 | 3.39 | 0.70 | 0.02 |
|  |  | Trained 2 | Clinic | 1 | 9.82 | 1.56 | 0.00 | 0.00 | 36.5 | 4.71 | 0.29 | 0.06 |
|  |  |  | Home | 1 | 9.06 | 0.75 | 0.00 | 0.00 | 35.0 | 9.03 | 0.64 | 0.21 |
|  |  |  | Clinic | 10 | 9.79 | 0.61 | 0.03 | 0.01 | 33.6 | 6.62 | 0.75 | 0.05 |
|  |  |  | Home | 10 | 8.67 | 1.51 | 0.00 | 0.00 | 46.4 | 9.84 | 0.73 | 0.07 |
|  |  | Trained 3 | Clinic | 1 | 8.50 | 1.52 | 0.55 | 0.12 | 20.0 | 0.00 | 0.89 | 0.05 |
|  |  |  | Home | 1 | 10.16 | 2.38 | 0.45 | 0.22 | 55.0 | 14.43 | 0.94 | 0.02 |
|  |  |  | Clinic | 10 | 22.6 | 2.41 | 0.27 | 0.09 | 38.1 | 4.31 | 0.90 | 0.01 |
|  |  |  | Home | 10 | 23.0 | 1.30 | 0.14 | 0.03 | 35.9 | 2.38 | 0.93 | 0.01 |
| RH+SI | Untrained | Untrained 1 | Clinic | 1 | 14.81 | 5.04 | 0.84 | 0.46 | 8.00 | 0.00 | 0.56 | 0.13 |
|  |  |  | Home | 1 | NA | NA | NA | NA | NA | NA | NA | NA |
|  |  |  | Clinic | 10 | 10.66 | 0.84 | 1.46 | 0.11 | 13.7 | 1.05 | 0.64 | 0.03 |
|  |  |  | Home | 10 | 8.90 | 0.41 | 1.53 | 0.06 | 14.2 | 0.29 | 0.63 | 0.02 |
|  |  | Untrained 2 | Clinic | 1 | 9.72 | 0.56 | 0.50 | 0.13 | 118 | 2.00 | 0.96 | 0.01 |
|  |  |  | Home | 1 | 11.7 | 1.92 | 0.40 | 0.07 | 26.0 | 8.25 | 0.97 | 0.02 |
|  |  |  | Clinic | 10 | 20.4 | 3.07 | 0.40 | 0.06 | 41.7 | 8.10 | 0.94 | 0.02 |
|  |  |  | Home | 10 | 24.9 | 3.56 | 0.31 | 0.04 | 29.9 | 2.51 | 0.95 | 0.01 |
|  |  | Untrained 3 | Clinic | 1 | 12.1 | 0.26 | 1.02 | 0.71 | 55.0 | 0.00 | 0.60 | 0.09 |
|  |  |  | Home | 1 | 10.3 | 0.20 | 1.07 | 1.07 | 15.0 | 0.00 | 0.47 | 0.00 |
|  |  |  | Clinic | 10 | 11.2 | 0.71 | 1.38 | 0.14 | 76.1 | 6.14 | 0.58 | 0.04 |
|  |  |  | Home | 10 | 9.06 | 0.76 | 1.27 | 0.10 | 20.7 | 1.47 | 0.50 | 0.02 |
|  | Trained | Trained 1 | Clinic | 1 | 17.9 | 12.50 | 0.24 | 0.04 | 33.0 | 9.54 | 0.52 | 0.05 |
|  |  |  | Home | 1 | 21.9 | 15.26 | 0.06 | 0.06 | 54.4 | 10.15 | 0.54 | 0.07 |
|  |  |  | Clinic | 10 | 19.9 | 3.28 | 0.64 | 0.07 | 51.1 | 4.97 | 0.60 | 0.02 |
|  |  |  | Home | 10 | 11.1 | 2.34 | 0.42 | 0.06 | 51.4 | 3.39 | 0.60 | 0.02 |
|  |  | Trained 2 | Clinic | 1 | 14.1 | 4.02 | 0.33 | 0.29 | 19.3 | 3.61 | 0.83 | 0.07 |
|  |  |  | Home | 1 | 15.8 | 5.50 | 2.09 | 0.51 | 20.0 | 0.00 | 0.86 | 0.07 |
|  |  |  | Clinic | 10 | 9.84 | 1.29 | 0.45 | 0.07 | 15.4 | 1.89 | 0.78 | 0.05 |
|  |  |  | Home | 10 | 12.2 | 2.27 | 0.37 | 0.14 | 19.3 | 6.54 | 0.76 | 0.06 |
|  |  | Trained 3 | Clinic | 1 | 19.8 | 5.17 | 2.01 | 0.27 | 35.0 | 7.79 | 0.91 | 0.04 |
|  |  |  | Home | 1 | 23.4 | 9.44 | 1.93 | 0.28 | 46.7 | 10.54 | 0.92 | 0.03 |
|  |  |  | Clinic | 10 | 41.4 | 4.57 | 0.83 | 0.21 | 31.8 | 3.61 | 0.83 | 0.02 |
|  |  |  | Home | 10 | 46.2 | 3.20 | 0.68 | 0.12 | 28.2 | 1.88 | 0.82 | 0.01 |

**APPENDIX B**

Plots of response latency by task, location, and group.

**
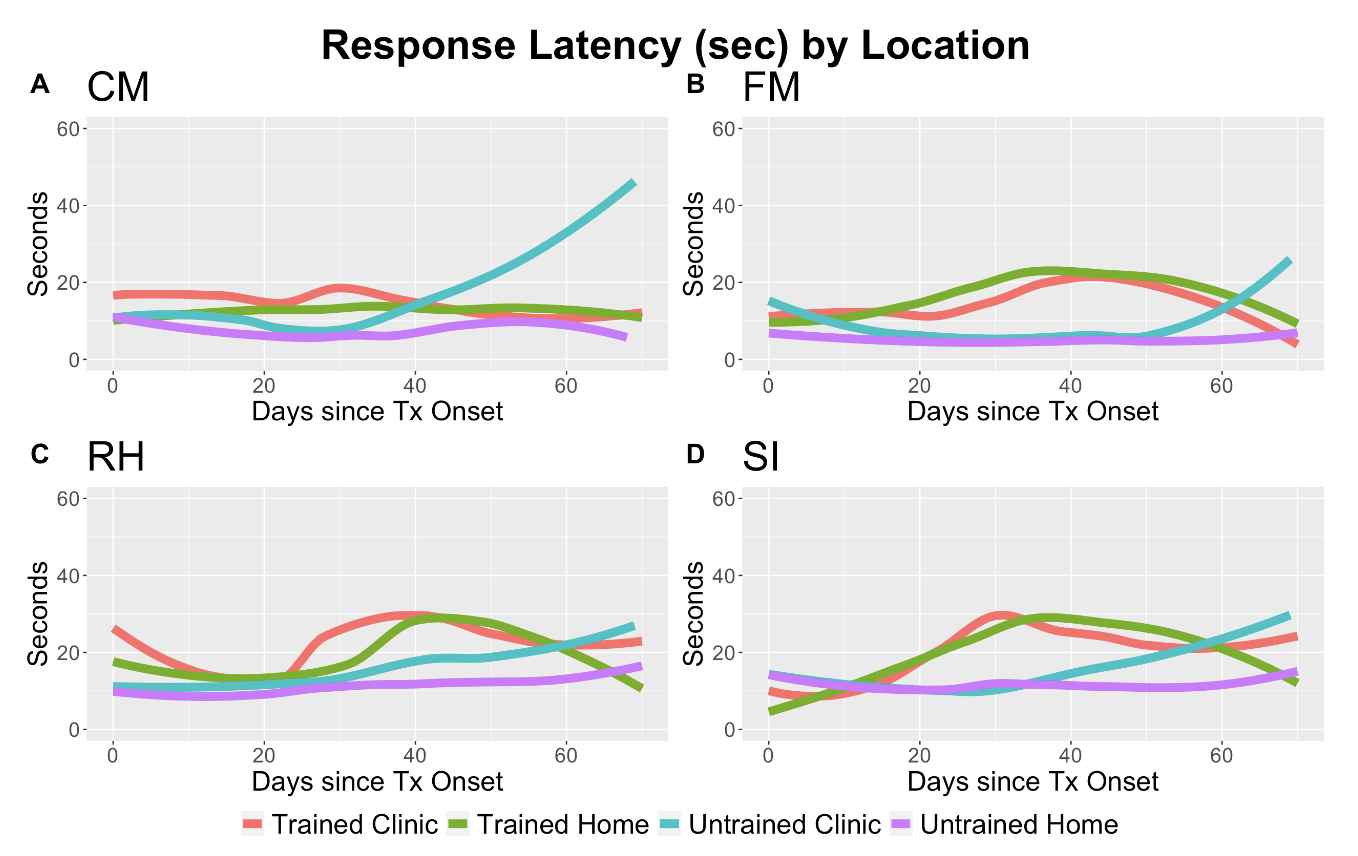
**

**APPENDIX C**

Plots of response latency by task, location, and group.

**
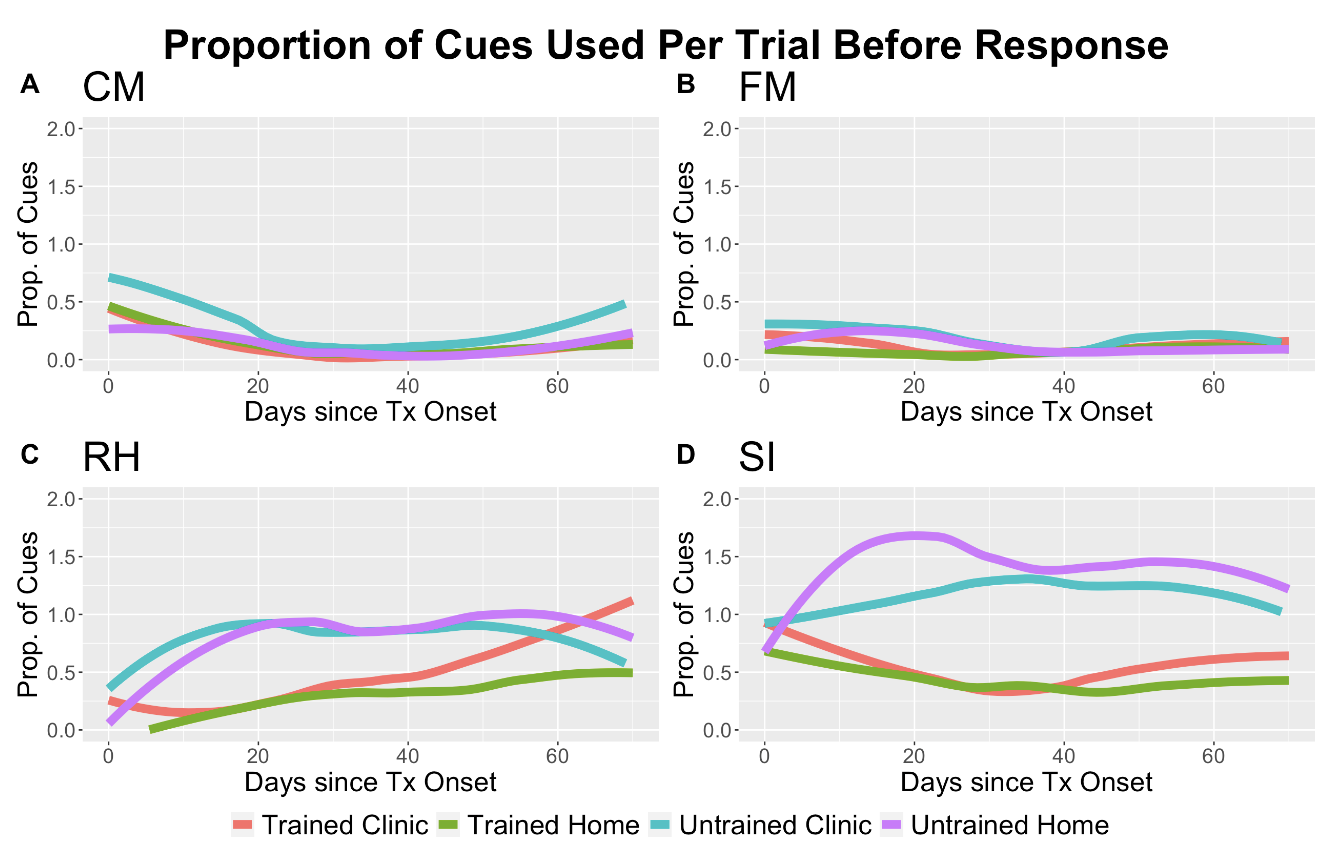
**

**SUPPLEMENTARY MATERIALS**

**Treatment Protocol – Trained Group: Instructions for Clinicians**

The goal of this treatment protocol is to teach participants how to better manage feedback and to deepen the depth of their processing when engaging with constant therapy. The goal is to 1) identify challenges presented by each therapy task, 2) develop and apply a planned approach for formulating responses and managing task demands, and 3) turn incorrect trials into additional learning opportunities.

Steps one and two are based on meta-cognitive strategy training programs primarily implemented in populations with cognitive deficits (Kennedy et al., 2008; McEwen, Polatajko, Huijbregts, & Ryan, 2009; Skidmore et al., 2011). Such training encourages active engagement with therapy and is thought to promote increased awareness and monitoring. For step 3, anytime a response is skipped or incorrect, participants will be instructed to use cues integrated within Constant Therapy that allow for review of a target item or correct response. They will be encouraged to repeat the correct response prior to moving on.

*In a nutshell, what this means for you:*

- 1. Maximize naming opportunities. A client should be encouraged to name a picture anytime they see one.
  2. Many clients immediately press buttons in the app that gives them the name of the item. Encourage clients to try to retrieve the name independently, and based on what they think the word is, make their response.
  3. Once they have responded, they can use a cue to check if they are correct or hear the item name.
  4. Encourage participant to repeat the name of the pictured item after hearing the cue.
  5. Anytime a response is incorrect or uncertain, you will train participants to elicit feedback and cues from the app.

It will be tempting to provide semantic cues to participants or to use language strategies to help them overcome difficulties. It is very important for the study protocol, however, that your instruction remains focused on guidance to practice naming, elicit and manage feedback. The research question is twofold:

1. Can participants learn to independently practice naming, checking responses vie cues provided in the app?
2. Does reviewing responses after errors improve treatment outcomes with constant therapy?

**Task Descriptions**

| Category Matching | Yes/no questions about whether two written words belong in the same category or not |
| --- | --- |
| Syllable Identification | Decide whether the name of a picture has 2 syllables |
| Feature Matching | Yes/no questions about characteristics of an image |
| Rhyming | Yes/no questions about whether the name of the image presented rhymes with a given word |

**Start of session**

Start each session by touching base about app use and any questions/difficulty with the iPad.

1. I see that you logged in X times since I last saw you.

a. If they’ve logged in every day or nearly every day: Great. I’m glad to see that you’re using the app frequently.

b. If they haven’t logged in frequently: I see that you didn’t log in very much. What happened?

2. Did you have any trouble using the iPad or logging in to Constant Therapy?

3. Is there any task that you feel is particularly difficult?

**Session**

Ask the participant to login to Constant Therapy to do their tasks

• Try to have the participant do this as independently as possible. Note any difficulties and provide guidance for logging in.

• Please be sure that the participant does all of the button pressing/typing to gain practice logging into the app.

Whenever the participant makes an error or goes to skip a question:

• “Let’s see if we can figure out the answer”

• “Pause on this screen. See that button there? Pressing that lets you hear the correct answer.”

• “Press the cue and then try to say the word while you focus on the picture.”

**Data collection:**

• Please write down the target item

• If correct on the first try: Make a check in the Correct column

• If correct after multiple tries: in the Incorrect column, write a number indicating how many tries it took to reach a correct response.

- For example, if first guess is incorrect, next try is correct, write 2 in Incorrect column.

**Task-Specific Instructions**

**Category Matching**


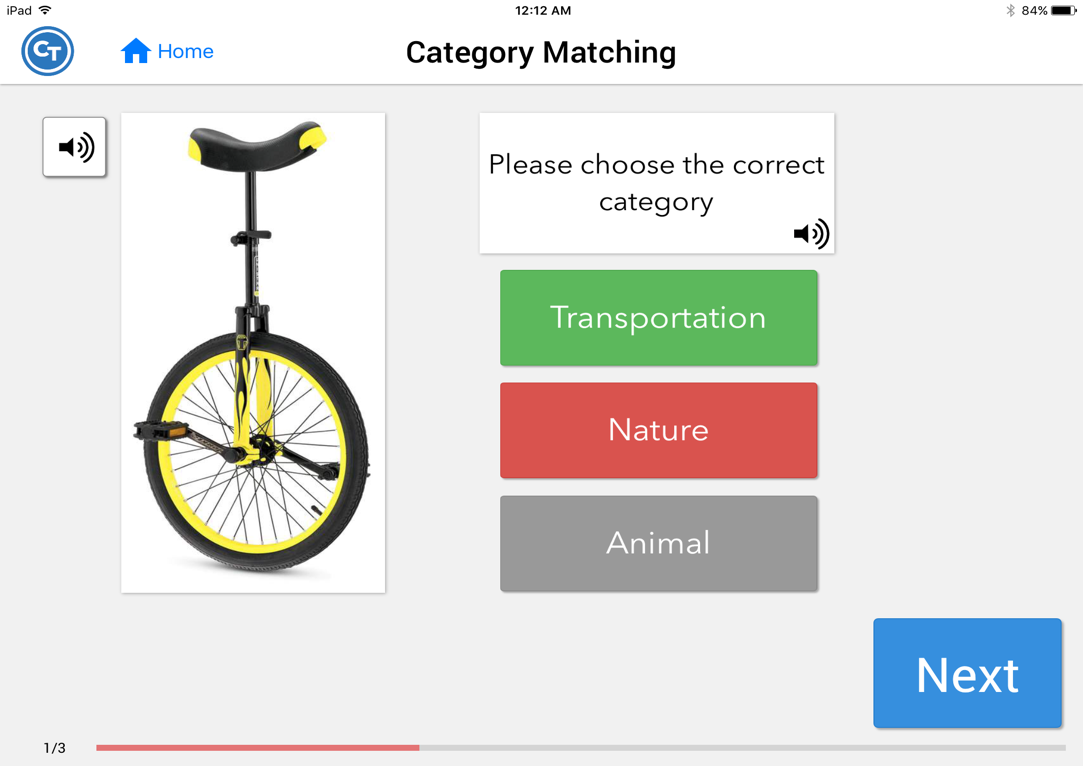


Encourage the participant to make a response based on what they think is the best answer.

AFTER they respond, ask: “what is that?”

If they don’t know or make a response and are uncertain, cue with: “Press this to hear the name of the pictured item.”

You may clarify: The correct category is highlighted in **green**. You may also provide a definition of a category.

Try to repeat the name of the item. Think about what it means to be in the category transportation.

**Feature Matching**


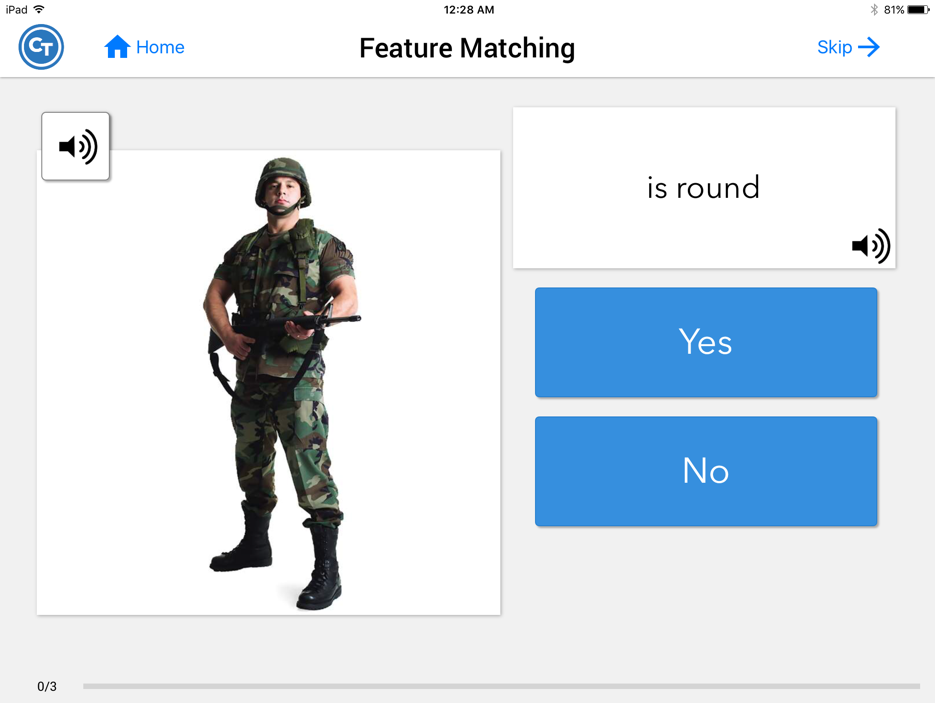


Encourage the participant to make a response based on what they think is the best answer.

AFTER they respond: Ask: “what is that?”

If they don’t know or make a response and are uncertain, cue with: “Press this to hear the name of the pictured item.”

If they ask you questions, direct them to use the cues available and to think through the correct answer.

NOTE: In this task, items often repeat.

**Rhyming**


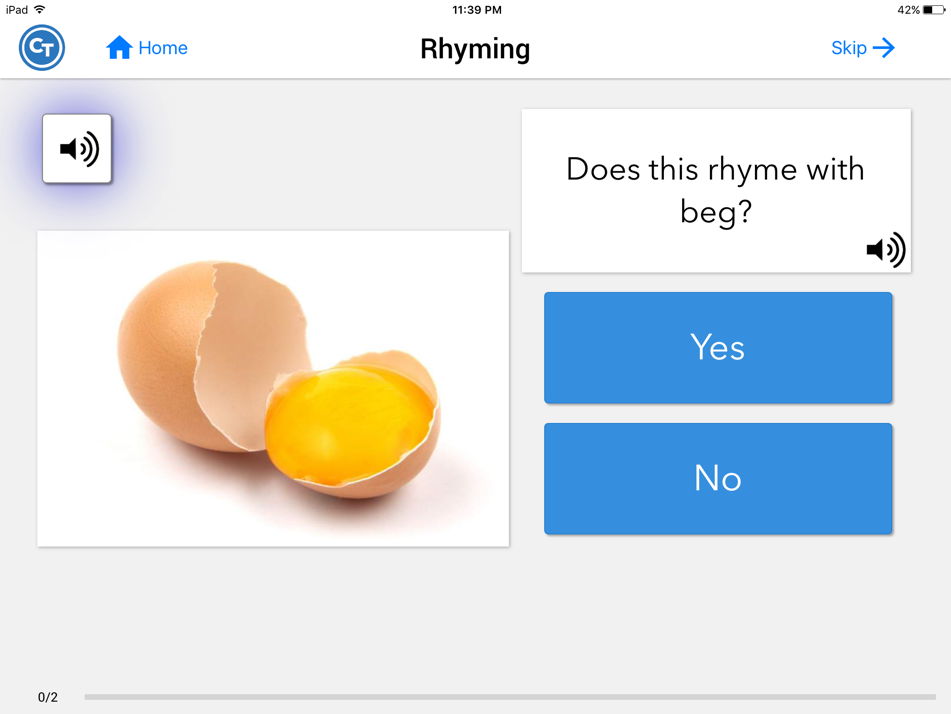


At the start and periodically throughout this task, you may review rhyming. “Remember rhyming words sound the same at the end.” Provide examples of some words that rhyme and some words that don’t rhyme. You may pick words similar to those presented on the screen, but do not use the target item as your example. First encourage the client to think of the name of the pictured item. See if they can think of the name before requesting the name from the app.

If they ask for help, state :“Based on what you think the name is, do you think those rhyme? What’s your best guess?”

**After making a response, or if the client is unable to respond without cues, state:** “Press this to hear the name of the pictured item. “ Encourage the participant to repeat the name.  **Remind them:** “Remember rhyming words sound the same at the end.”

**Syllable Identification**


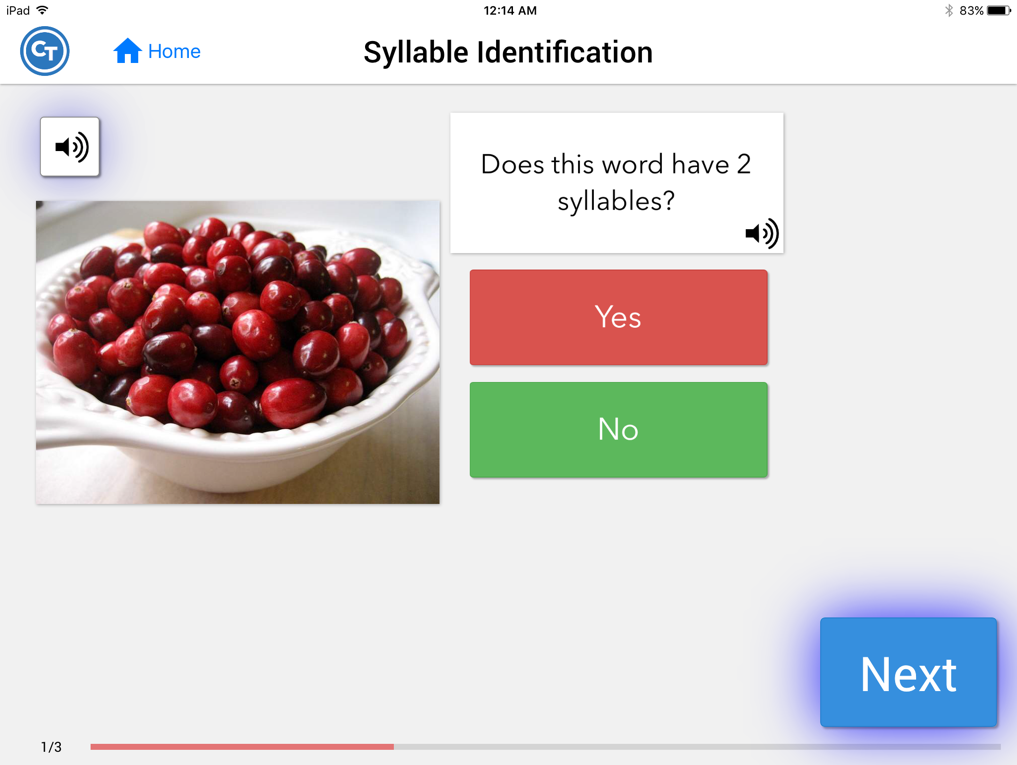


At the start of this task and periodically throughout you may work on syllable counting. Practice saying words and tapping out the number of syllables. You may tap out syllables to multisyllable words, not just 2-syllable words. Pick a word that is not presented on the screen. You can select a similar word, but not the word presented on the screen. First encourage the client to think of the name of the pictured item. See if they can think of the name before requesting the name from the app.

If unable to respond, ask the client: “Based on what you think the name is, do you think it has two syllables? What’s your best guess?”

After making a response, or if the client is unable to respond without cues, state: “Press this to hear the name of the pictured item.” Encourage the participant to repeat the name. You may also ask: “Does that have two syllables?” (you may repeat the word with the client) Encourage the participant to tap out the syllables independently.
